# Vernalization Requirement and the Chromosomal *VRN1*-Region can Affect Freezing Tolerance and Expression of Cold-Regulated Genes in *Festuca pratensis*

**DOI:** 10.3389/fpls.2016.00207

**Published:** 2016-02-25

**Authors:** Åshild Ergon, Tone I. Melby, Mats Höglind, Odd A. Rognli

**Affiliations:** ^1^Department of Plant Sciences, Norwegian University of Life SciencesKlepp Stasjon, Norway; ^2^Food and Agriculture Division, Norwegian Institute of Bioeconomy ResearchKlepp Stasjon, Norway

**Keywords:** *CBF6*, *COR14B*, *CR7*, deacclimation, meadow fescue, *IRI1*, *LOS2*, photoperiod

## Abstract

Plants adapted to cold winters go through annual cycles of gain followed by loss of freezing tolerance (cold acclimation and deacclimation). Warm spells during winter and early spring can cause deacclimation, and if temperatures drop, freezing damage may occur. Many plants are vernalized during winter, a process making them competent to flower in the following summer. In winter cereals, a coincidence in the timing of vernalization saturation, deacclimation, downregulation of cold-induced genes, and reduced ability to reacclimate, occurs under long photoperiods and is under control of the main regulator of vernalization requirement in cereals, *VRN1*, and/or closely linked gene(s). Thus, the probability of freezing damage after a warm spell may depend on both vernalization saturation and photoperiod. We investigated the role of vernalization and the *VRN1*-region on freezing tolerance of meadow fescue (*Festuca pratensis* Huds.), a perennial grass species. Two F_2_ populations, divergently selected for high and low vernalization requirement, were studied. Each genotype was characterized for the copy number of one of the four parental haplotypes of the *VRN1*-region. Clonal plants were cold acclimated for 2 weeks or vernalized/cold acclimated for a total of 9 weeks, after which the F_2_ populations reached different levels of vernalization saturation. Vernalized and cold acclimated plants were deacclimated for 1 week and then reacclimated for 2 weeks. All treatments were given at 8 h photoperiod. Flowering response, freezing tolerance and expression of the cold-induced genes *VRN1, MADS3, CBF6, COR14B, CR7* (*BLT14*), *LOS2*, and *IRI1* was measured. We found that some genotypes can lose some freezing tolerance after vernalization and a deacclimation–reacclimation cycle. The relationship between vernalization and freezing tolerance was complex. We found effects of the *VRN1*-region on freezing tolerance in plants cold acclimated for 2 weeks, timing of heading after 9 weeks of vernalization, expression of *COR14B, CBF6*, and *LOS2* in vernalized and/or deacclimated treatments, and restoration of freezing tolerance during reacclimation. While expression of *VRN1, COR14B, CBF6, LOS2*, and *IRI1* was correlated, *CR7* was associated with vernalization requirement by other mechanisms, and appeared to play a role in freezing tolerance in reacclimated plants.

## Introduction

Overwintering temperate plants cold acclimate in the autumn and develop resistance to freezing damage. Upon exposure to warmer temperatures in spring plants deacclimate and gradually lose this resistance (reviewed by Kalberer et al., 2006; Rapacz et al., 2014). Warm spells in mid-winter or early spring can cause deacclimation when there is still a risk of freezing temperatures. To some extent, depending on circumstances, plants have the ability to reacclimate if temperatures drop again. The annual variation in freezing tolerance is one of many developmental processes that are regulated largely by temperature (Penfield, 2008). Deacclimation and reacclimation processes are highly complex, and although temperature is a main driving force, other environmental and physiological conditions have strong influence. Resistance to deacclimation and/or the ability to reacclimate is thought to be crucial for plant winter survival in areas with a variable winter climate and temperatures fluctuating around the freezing point. In the face of global warming, where a higher frequency of warm spells during winter can be expected (Shabbar and Bonsal, 2003; Johansson et al., 2011), understanding these processes in plants is important both in an agricultural and an ecological context (Gu et al., 2008; Bokhorst et al., 2009; Rapacz et al., 2014).

The relationships between vernalization (the process of becoming competent to flower after a prolonged period of cold) and freezing tolerance, and to some extent photoperiod, have been particularly studied in cereals. In these species, it has been shown that freezing tolerance and expression of genes involved in freezing tolerance are down-regulated in leaf and stem base tissue when the vernalization requirement is saturated, but before any development of the apex is visible in the microscope (Fowler et al., 1996; Limin and Fowler, 2006; Laudencia-Chingcuanco et al., 2011). There is an interaction between vernalization and photoperiod on this deacclimation and also on the ability to reacclimate. In cultivars with a long day requirement for flowering, the negative effect of vernalization on freezing tolerance is stronger when plants are vernalized under long days than under short days, whereas vernalization- and photoperiod-insensitive cultivars are not able to develop much freezing tolerance at all (Mahfoozi et al., 2001a, 2005, 2006; Dhillon et al., 2010). Also, plants vernalized and deacclimated under long days are less able to reacclimate (Mahfoozi et al., 2001b). *VRN1* is an inducer of the transition to generative development in cereals and other temperate grass species (reviewed by Trevaskis, 2010; Fjellheim et al., 2014). It is gradually upregulated during vernalization and appears to act in the down-regulation of freezing tolerance genes in vernalized plants under long days (Fowler et al., 1996; Limin and Fowler, 2006; Dhillon et al., 2010; Laudencia-Chingcuanco et al., 2011). It is not entirely clear, however, whether it is *VRN1* itself, or a very closely linked gene, that is responsible. It is also not known how long days interact with the *VRN1* locus in down-regulation of freezing tolerance genes. Mahfoozi et al. (2005, 2006) suggested that in regions with long, mild winters, mechanisms extending the vegetative phase (through vernalization and/or photoperiod requirements) might actually be more important for winter survival than a high maximum attainable freezing tolerance. This is increasingly relevant for winter cereals and perennial grasses in the context of climate change in Northern areas, where winters will become milder, but remain dark.

There are several reports describing deacclimation and reacclimation in response to various treatments in perennial grass species (Tronsmo, 1985; Gay and Eagles, 1991; Tompkins et al., 2000; Jørgensen et al., 2010; Espevig et al., 2014; Hoffmann et al., 2014). These have, however, not specifically tested the effect of vernalization or vernalization requirement on deacclimation and reacclimation, or characterized accompanying changes in gene expression. Here, we addressed these aspects by studying freezing tolerance during a cold acclimation (CA)/vernalization – deacclimation – reacclimation cycle in genetic material of the perennial forage grass species *Festuca pratensis* Huds. (meadow fescue), divergently selected for high or low vernalization requirement. We coupled this with measurements of flowering response and expression of *VRN1* and *MADS3* (a *VRN1*-like gene), and genes known to play a role in CA in *F. pratensis* and other temperate grass species (**Table 1**).

**Table 1:** Genes included in the gene expression analysis.

Gene	Protein	Expression	Function	Reference
*VRN1*	MADS-box transcription factor	Induced by prolonged cold. Induced by long photoperiod under certain circumstances.	Induces transition to generative development	Yan et al., 2003; Jensen et al., 2005; Ergon et al., 2006; Trevaskis, 2010
*MADS3*	VRN1-like MADS-box transcription factor	Associated with transition to generative development	Unknown	Schmitz et al., 2000; Petersen et al., 2004; Preston and Kellogg, 2008; Ergon et al., 2013
*CBF6*	Member of family of AP2/EREBP transcription factors	Induced rapidly by cold	Induces cold-regulated genes with CRT/DRE promoter element.	Xiong and Fei, 2006; Tamura and Yamada, 2007; Galiba et al., 2009; Alm et al., 2011; Rudi et al., 2011; Sandve et al., 2011; Jurczyk et al., 2013a
*COR14B*	Soluble protein localized in the stroma compartment of the chloroplast	Induced by *CBF*s. Regulated by light. Some expression also in etiolated tissue and stem base.	Unknown	Crosatti et al., 1995, 1999, 2003; Dal Bosco et al., 2003; Galiba et al., 2009; Rudi et al., 2011; Jurczyk et al., 2013a
*CR7 (BLT14)*	Member of a family of proteins predicted to be secreted into the apoplast	Cold-induced	Unknown	Phillips et al., 1997; Pearce et al., 1998; Rudi et al., 2011
*LOS2*	Bifunctional enolase and transcription factor	Cold-induced	Positive regulator of cold-induced genes	Lee et al., 2002; Rudi et al., 2011; Jurczyk et al., 2013a
*IRI1*	Member of a family of ice recrystallization inhibitor proteins	Cold-induced	Protects against freezing damage	Tremblay et al., 2005; Sandve et al., 2008; Zhang et al., 2010; Rudi et al., 2011

## Materials and Methods

### Plant Material and Growth Conditions

**FIGURE 1:**
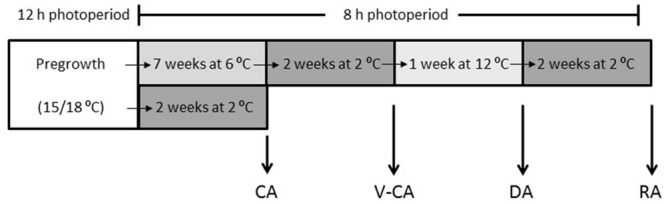
**Overview of the four different temperature treatments.** Clonal plants of the VRmin and VRmax F_2_ populations of *Festuca pratensis*, divergently selected for low and high vernalization requirement, respectively, were pregrown at 12 h photoperiod and 15/18°C and then exposed to four different temperature treatments: **CA:** cold acclimation at 2°C for 2 weeks, **V-CA:** vernalization and cold acclimation at 6°C for 7 weeks + 2°C for 2 weeks, **DA:** V-CA followed by deacclimation at 12°C for 1 week, **RA:** V-CA and DA followed by reacclimation for 2 weeks at 2°C, all at 8 h photoperiod. After each of the four temperature treatments plants were sampled for determination of flowering response, freezing tolerance, and gene expression.

Genotypes of two F_2_-populations from the “HF2/7 × BF14/16” F_1_ mapping population of *Festuca pratensis* Huds. (Alm et al., 2003), VRmin and VRmax, were studied. These two populations were produced by crossing F_1_ individuals selected for either high or low vernalization requirement in two separate groups (Ergon et al., 2013). VRmin segregates for the ability to flower without vernalization, while VRmax requires 9 or more weeks of vernalization in order to flower. Seeds were sown in November 2011 and the plants were grown in the greenhouse under non-vernalizing temperatures and 12 h photoperiod. Over the summer the pots were kept outdoors with natural light conditions. In late August 2012, twenty-one and six genotypes of VRmin and VRmax, respectively, were clonally propagated and pregrown in the greenhouse. A higher number of VRmin genotypes were included due to the segregation for the ability to flower without vernalization in this population. The plants were first grown at 15/18°C (day/night), 12 h photoperiod and approximately 100 μmol m^-2^s^-1^ PAR for 2 months and cut and fertilized at 1 or 2 weeks intervals. After this the plants were grown for another 2 months with approximately 200 μmol m^-2^s^-1^ PAR, and not cut, but fertilized weekly. After pregrowth, the plants were exposed to four different temperature treatments (**Figure 1**); CA at 2°C for 2 weeks, V-CA: vernalization and CA at 6°C for 7 weeks followed by 2 weeks at 2°C, DA: V-CA followed by deacclimation at 12°C for 1 week, RA: V-CA and DA followed by reacclimation for 2 weeks at 2°C. All treatments had a 8 h photoperiod and a light intensity of approximately 250 μmol m^-2^s^-1^ PAR. Throughout the experiment plants were organized into trays with one plant of each genotype, and random trays of plants were used for testing of freezing tolerance, flowering response and tissue sampling after each of the four temperature treatments.

### Determination of Flowering Response and Freezing Tolerance

For determination of the heading phenotype, three plants per genotype and temperature treatment were placed in the greenhouse at approximately 18°C and 16 h photoperiod. The flowering response was recorded as DTH (number of days from transfer to the greenhouse until the tip of the first panicle was visible), and as PHP (percent heading plants) and PHS (percent heading shoots per plant), both recorded when heading ceased (no new plants heading for a week).

For determination of freezing tolerance (LT50), four plants of each genotype and temperature treatment were divided into individual tillers. The shoots were cut at 5 cm and the roots at 2 cm and 4–9 random tillers from each genotype were placed in loose bundles in each of 13 boxes of moist sand in programmed freezing chambers initially set at 2°C. One control box was placed in a chamber with a constant temperature of 2°C, while the other 12 boxes were distributed among three chambers where the temperature was first lowered from 2°C to –3°C at 1°C h^-1^ and kept at this level for 12 h, after which the temperature was lowered again by 1°C h^-1^. Four test temperatures were used; –5, –10, –15, and –20°C for the CA treatment, and –13, –17, –21, and –25°C for the V-CA, DA, and RA treatments. When the temperature reached one of the four test temperatures, one box from each of the three chambers was removed and placed at 2°C for thawing. After thawing tillers were planted in soil. Survival of individual plants, rated dead or alive, was determined after 3 weeks of growth in a greenhouse at approximately 18°C and 16 h photoperiod. Freezing tolerance (LT50; temperature required to kill 50% of the tillers) was calculated by probit analysis using PROC PROBIT in SAS 9.2 (SAS Institute, Inc., Cary, NC, USA).

### Gene Expression Analysis

The shoot bases (1 cm, outer leaves peeled off) of all tillers from one plant per genotype and temperature treatment were excised, immediately frozen in liquid nitrogen and then kept at –80°C. Sampling was done during a 3 h period from 1 to 4 h after dawn, and the genotypes were sampled in a random order. Total RNA was extracted with the RNeasy Plant Mini kit (Qiagen). Ten microgram RNA of each sample was DNAse-treated with TURBO DNase (Ambion, Life Technologies) and 1 μg of DNAse-treated RNA was used as a template for cDNA-synthesis using the SuperScript VILO cDNA synthesis kit (Invitrogen, Life Technologies). A 10 μl control reaction without reverse transcriptase was included for all samples in order to confirm the absence of genomic DNA contamination. All cDNA samples were diluted 5x and 2 μl was used as template in real-time PCR reactions with SYBRGreen PCR Master Mix (Applied Biosystems, Life Technologies) in order to quantify transcript levels of *VRN1, MADS3, CBF6, COR14B, CR7, LOS2*, and *IRI1* (see Supplementary Table S1 for primer sequences). The house-keeping gene *ACTIN* was used as a reference gene. PCR products were quantified in a 7500 Fast Real-Time PCR system (Applied Biosystems, Life Technologies). Relative quantity (RQ) of transcripts in each sample was determined by the Δ*C*_t_-method, where RQ = 2^-Δ^*^Ct^* and Δ*C*_t_ = *C*_tgeneofinterest_ – *C*_tACTIN_. Twenty-seven of the 756 sample-gene combinations, for which the qRT-PCR were not successful, were regarded as missing values in the analyses. In addition, four sample-gene combinations with more than 3x higher expression than the other samples in the same temperature treatment were considered as outliers and also regarded as missing values.

### Classification into Phenotypic and Genotypic Classes

Based on the ability to head or not without vernalization (after CA only), the genotypes of VRmin were divided into two phenotypic classes: VRmin- (able to head without vernalization, seven genotypes) and VRmin+ (unable to head without vernalization, 14 genotypes). The *VRN1*-locus of all individuals was genotyped using the CAPS-marker described by Ergon et al. (2006), which recognizes one of the four haplotypes (*b*) of the *VRN1*-locus in this population. This haplotype is one of the two maternal haplotypes of a region of chromosome four containing QTLs controlling vernalization requirement (Ergon et al., 2006) and freezing tolerance (Alm et al., 2011) in the F_1_ mapping population. The *b*-haplotype is associated with low vernalization requirement and high freezing tolerance. Based on the *b*-allele, individuals in the VRmin population were divided into three genotypic classes: homozygous for the *b*-allele (four genotypes), heterozygous (nine genotypes), or no *b*-allele (eight genotypes). VRmax was not divided into genotypic classes due to the limited number of genotypes included from this population (*bb*:1, –*b*:3, –:2).

### Statistical Analysis

To test for differences between F_2_ populations and phenotypic and genotypic classes of VRmin, the flowering response, freezing tolerance and gene expression data were subjected to analysis of variance using PROC GLM procedure in SAS 9.2. Pairwise differences between treatments were identified with the LSD-test of GLM. PROC CORR procedure was used to test for correlation among all traits of VRmin genotypes within temperature treatments.

## Results

### Flowering Response

**FIGURE 2:**
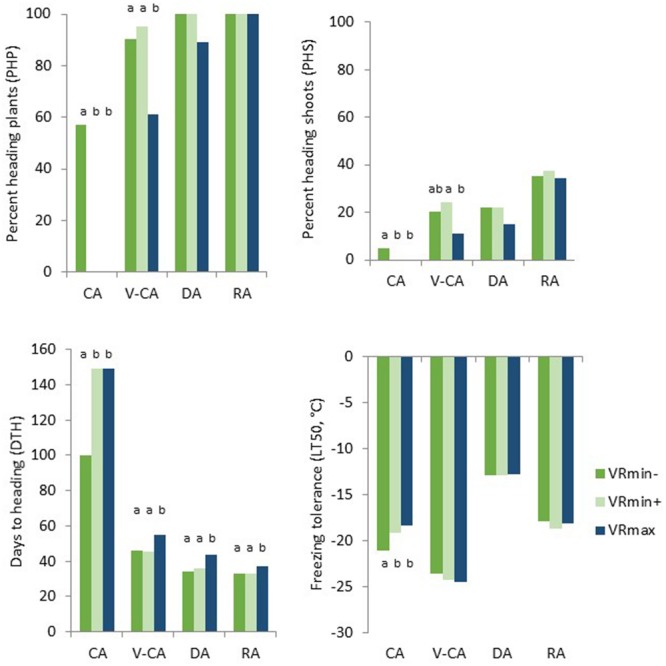
**Heading response (measured as percent heading plants, percent heading shoots per plant and days to heading) and freezing tolerance of clonal plants of *Festuca pratensis* genotypes exposed to four different temperature treatments, CA, V-CA, DA, and RA (see **Figure 1**).** Genotypes of two F_2_ populations selected for either high (**VRmax**, six genotypes) or low (VRmin) vernalisation requirement were tested; VRmin were further divided into two phenotypic classes (**VRmin–**, seven genotypes, and **VRmin+**, 14 genotypes), based on the heading response in CA. Plants that did not head during the experiment were given the value 149 for days to heading. LT50: freezing temperature at which 50% of the plants were estimated to die. Significant differences between groups within temperature treatments (*P* < 0.05) are indicated with different letters above bars.

**FIGURE 3:**
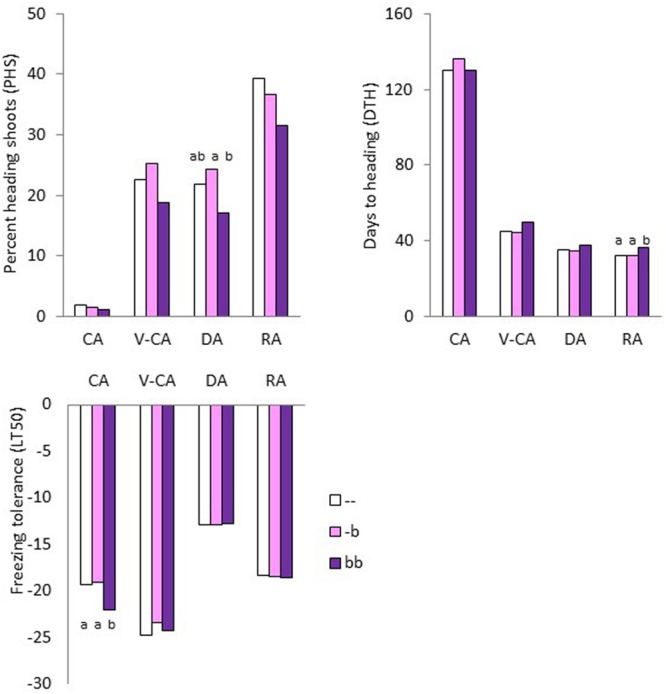
**Heading response (measured as percent heading shoots per plant and days to heading) and freezing tolerance of clonal plants of 21 genotypes in the VRmin F_2_ population of *Festuca pratensis*, exposed to four different temperature treatments, CA, V-CA, DA, and RA (see **Figure 1**).** The genotypes were classified according to the number of copies of the *b*-haplotype of the *VRN1* chromosomal region. *N* = 8 (–), 9 (-*b*), and 4 (*bb*). Plants that did not head during the experiment were given the value 149 for days to heading. LT50, freezing temperature at which 50% of the plants were estimated to die. Significant differences between groups within temperature treatments (*P* < 0.05) are indicated with different letters above bars. There were no differences between genotypic classes in percent of heading plants (not shown).

The heading response differed significantly between the two F_2_ populations and between the four temperature treatments (**Table 2**). Some VRmin genotypes (defined as phenotypic class VRmin-) headed sparsely after only 2 weeks of CA followed by greenhouse conditions (**Figure 2**). VRmin- genotypes did not differ significantly from VRmin+ in the other three temperature treatments. After a total of 9 weeks at low temperatures (V-CA), VRmin genotypes had an average PHP of 94, PHS of 23 and DTH of 46 (**Table 3**). VRmax genotypes headed significantly less and later with PHP, PHS and DTH values of 61, 11, and 55, respectively. When V-CA plants were exposed to 1 week of de-acclimation (DA) the PHP and PHS did not change significantly, and both populations headed approximately 10 days earlier, indicating that the process of heading started during the DA treatment. When DA plants were exposed to 2 weeks of reacclimation (RA) all the plants of both populations headed (PHP = 100%). Both populations obtained higher PHS than V-CA and DA plants and headed earlier than V-CA plants. This response was stronger in VRmax genotypes and a significant difference between the populations remained for DTH only. After RA, the PHP and PHS values had changed by 6 and 64% relative to the values after the V-CA treatment in VRmin, while in VRmax, they changed by 64 and 209%, respectively. Thus, the vernalization requirement was not saturated after 9 weeks of low temperature in any of the populations, but VRmin was closer to saturation than VRmax. After V-CA, DA and RA, VRmin genotypes homozygous for the *b*-haplotype of the *VRN1-*region tended to head later and have lower PHS than other genotypes, although this was not significant for all treatments (**Figure 3**).

**Table 2:** Results from analysis of variance using the model *Variable = Temperature treatment (T)* + *Population (P)* + *T* × *P* + *Error*.

Variable	Temperature treatment (T)	Population (P)	*T* × *P*
	*d.f*. = 3	*d.f*. = 1	*d.f*. = 3
PHP	114.5^∗∗∗^	12.0^∗∗∗^	NS
PHS	74.6^∗∗∗^	8.6^∗∗^	NS
DTH	399.7^∗∗∗^	10.7^∗∗^	NS
LT50	221.5^∗∗∗^	NS	NS
*CBF6*	3.71^∗^	NS	NS
*COR14B*	7.11^∗∗∗^	NS	NS
*CR7*	19.0^∗∗∗^	13.8^∗∗∗^	NS
*LOS2*	100.1^∗∗∗^	NS	NS
*IRI1*	9.0^∗∗∗^	NS	NS
*VRN1*	50.6^∗∗∗^	NS	NS
*MADS3*	19.0^∗∗∗^	NS	NS

*d.f., degrees of freedom; PHP, percent of heading plants; PHS, percent of heading shoots per plant; DTH, days to heading; LT50, freezing temperature at which 50% of the plants are estimated to die according to results from a freezing test. Remaining variables are expression levels (RQ) of various genes in the shoot base tissue. *F*-values are given when the effect was significant. ^∗∗∗^*P* < 0.001; ^∗∗^0.01 < *P* < 0.001; ^∗^0.01 < *P* < 0.05; NS, 0.05 < *P*.*

**Table 3:** Percentage of heading plants (PHP), percent heading shoots per plant (PHS), days to heading (DTH) and freezing tolerance (LT50, the temperature at which 50% of the plants are estimated to die) in two F_2_-populations, VRmin (21 genotypes) and VRmax (six genotypes), divergently selected for vernalization requirement from the *Festuca pratensis* F_1_ mapping family ‘B14/16 × HF2/7’.

Trait	Population	Temperature treatment			
		CA	V-CA	DA	RA
PHP	VRmin	19 ± 7 (0–100)^b^	94 ± 3 (67-100)^Aa^	100^Aa^	100^a^
	VRmax	0 ^c^	61 ± 20 (0-100)^Bb^	89 ± 11 (33-100)^Bab^	100^a^
PHS	VRmin	2 ± 1 (0–9)^c^	23 ± 2 (6–37)^Ab^	22 ± 1 (11–31)^Ab^	37 ± 3 (16–55)^a^
	VRmax	0^c^	11 ± 5 (0–30)^Bbc^	15 ± 4 (4–29)^Bb^	34 ± 6 (18–54)^a^
DTH^1^	VRmin	133 ± 6 (61–max^2^)^a^	46 ± 1 (41–56)^Bb^	35 ± 1 (32–42)^Bc^	33 ± 1 (29–38)^Bc^
	VRmax	Max^a^	55 ± 4 (41–66)^Ab^	44 ± 3 (36–51)^Ac^	37 ± 2 (32–42)^Ac^
LT50^3^	VRmin	–19.8 ± 0.5 (–15.2 to –26.1)^c^	–24.1 ± 0.4 (–19.5 to –28.0)^d^	–12.9 ± 0.04 (–12.6 to –13.2)^a^	–18.5 ± 0.3 (–14.5 to –21.0)^b^
	VRmax	–18.4 ± 0.7 (–15.4 to –19.4)^b^	–24.6 ± 0.8 (–21.8 to –26.6)^c^	–12.8 ± 0.07 (–12.6 to –13.0)^a^	–18.1 ± 0.4 (–17.1 to –19.7)^b^

*Clonal plants were exposed to four different temperature treatments (see **Figure 1**) before being tested for the heading response and freezing tolerance. For the heading characteristics there were three replicate plants per genotype and temperature treatment. For the LT50 test tillers were exposed to four test temperatures (4–10 tillers per test temperature in each of three replicate chambers). The values given are the averages of the genotypes ±SE, the genotype ranges are given in brackets. Values not followed by the same letter are significantly different according to one-way analyses of variance within the temperature treatments (capital letters) or within populations (small letters; *P* < 0.05).^1^DTH was measured from the time of transfer to long days and growing temperatures in a greenhouse. ^2^For plants that did not head during the course of the experiment the DTH was set to 149 days (the maximum DTH observed + 1). ^3^In treatment CA, there were only two replicate LT50 tests for 10 and 2 genotypes in VRmin and VRmax, respectively, due to a limited number of tillers available for testing. In treatment DA, LT50 could only be estimated for 17 and 4 genotypes in VRmin and VRmax, respectively, because there was 100% mortality in the other genotypes. The LT50 was for these were set at –12.6°C (the highest observed LT50 + 0.1) and the reported LT50 may therefore be an overestimate.*

### Freezing Tolerance

As expected, freezing tolerance differed between temperature treatments (**Tables 2** and **3**). After 2 weeks of CA LT50 was on average –19.5°C, while after a total of 9 weeks at low temperature it was –24.2°C. One week of de-acclimation lowered it to –12.8°C, and 2 weeks of re-acclimation raised it to –18.4°C, which was a significantly lower freezing tolerance than after the first 2 weeks of CA. There were no significant differences between populations within each temperature treatment. However, while VRmax was able to obtain the same level of freezing tolerance after RA as it had after CA, VRmin was not. This means that, on average, VRmin genotypes lost some ability to cold acclimate during vernalization and deacclimation, and this was caused by the VRmin- phenotypic class of VRmin (**Figure 2**). VRmin- was significantly more freezing tolerant than VRmin+ and VRmax in the CA treatment (LT50 –21.1, –19.1, and –18.4, respectively, *P* = 0.05). In the other treatments, there were no significant differences between populations or phenotypic classes. When grouping the VRmin genotypes into genotypic classes, those that were homozygous for the *b*-allele of the *VRN1*-region were more freezing tolerant than the other genotypes in the CA treatment (LT50 –22.0 and –19.2, respectively, *P* = 0.05, **Figure 3**).

### Gene Expression

**FIGURE 4:**
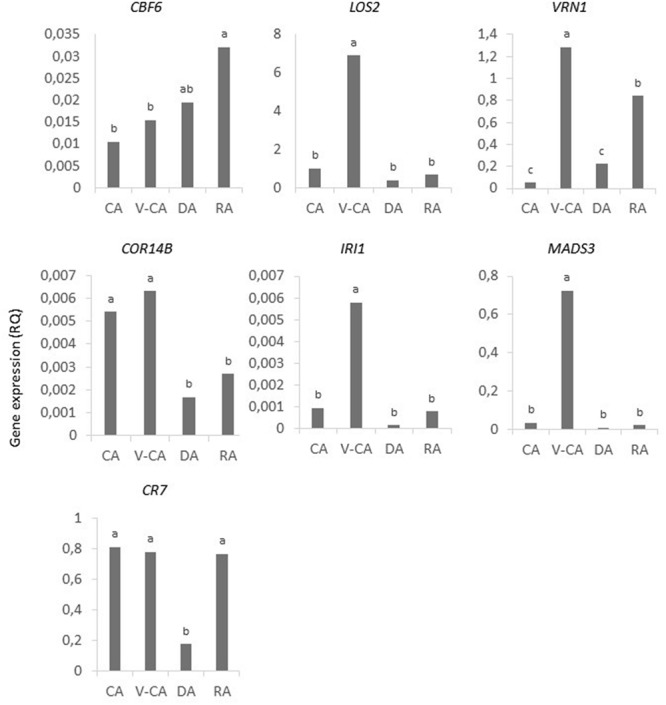
**Gene expression of *CBF6, LOS2, VRN1, COR14B, IRI1, MADS3*, and *CR7* in shoot bases of *Festuca pratensis* exposed to four different temperature treatments, CA, V-CA, DA, and RA (see **Figure 1**).** Average values of all 27 genotypes are shown. Gene expression was measured by qRT-PCR as the relative quantity (RQ) of transcripts using *ACTIN* as the reference gene. Different letters indicate significant differences among temperature treatments (*P* < 0.05). In a few cases 1–4 genotypes are not represented due to failed or outlier qPCR reactions.

**FIGURE 5:**
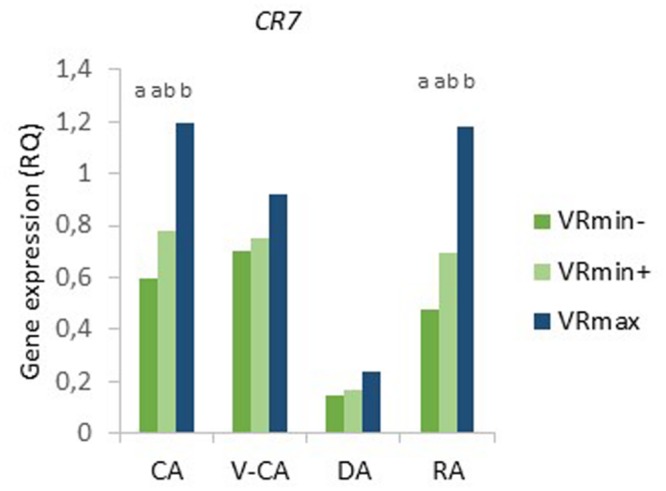
**Expression of *CR7* in shoot bases of *Festuca pratensis* exposed to four different temperature treatments, CA, V-CA, DA, and RA (see **Figure 1**).** Genotypes of two F_2_ populations selected for either high (**VRmax**, six genotypes) or low (VRmin) vernalisation requirement were tested; VRmin were further divided into two phenotypic classes (**VRmin–**, seven genotypes, and **VRmin+**, 14 genotypes), based on the heading response in CA. Gene expression was measured by qPCR as the RQ of transcripts using *ACTIN* as the reference gene. Different letters indicate significant differences within temperature treatments (*P* < 0.05). In two cases, two genotypes are not represented due to failed qPCR reactions.

**FIGURE 6:**
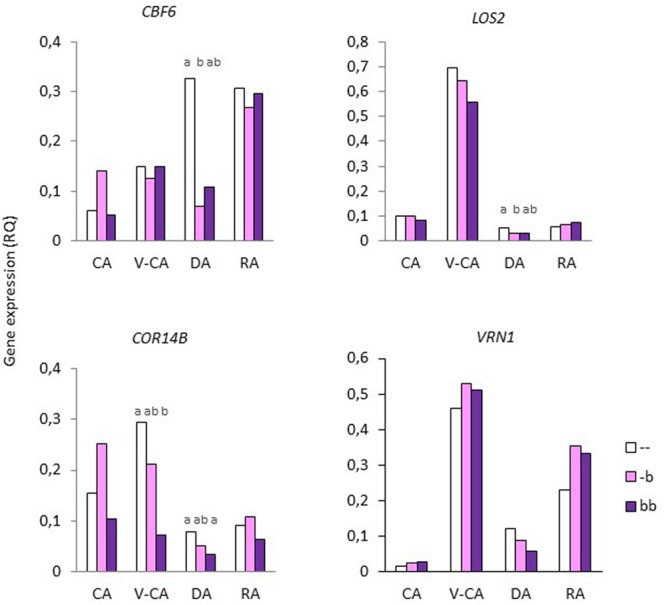
**Gene expression of *CBF6, LOS2, COR14B*, and *VRN1* in shoot bases of the *Festuca pratensis* VRmin population exposed to four different temperature treatments, CA, V-CA, DA, and RA (see **Figure 1**).** Plants were grouped according to the genotype identified by the *b*-haplotype of the *VRN1*-region. Gene expression was measured by qRT-PCR as the RQ of transcripts using *ACTIN* as the reference gene. Averages of 8 (–), 9 (-*b*), and 4 (*bb*) genotypes are shown. Different letters indicate significant differences within temperature treatments (*P* < 0.05). There were no differences between genotypic classes in the expression of *VRN1, MADS3, CR7*, or *LOS2* (only *VRN1* shown).

There was a significant effect of temperature treatment on expression of all genes (**Table 2**). The expression of *CBF6, COR14B*, and *CR7* were not significantly different after the V-CA treatment as compared to only CA treatment, while the expression of *VRN1* and *MADS3* was more than 20x higher, and *LOS2* and *IRI1* more than 5x higher, in the V-CA treatment than in the CA treatment (**Figure 4**). All genes except *CBF6* were downregulated by deacclimation. In DA, *LOS2, IRI1*, and *MADS3* were down-regulated to only 0.06x or less of the RQ in V-CA, while *VRN1, COR14B*, and *CR7* were down-regulated to around 0.25x of the RQ in V-CA (and CA in the case of *COR14B* and *CR7*). Only *VRN1* and *CR7* were significantly up-regulated by re-acclimation (4x higher RQ in RA than in DA). The expression of *VRN1* became 16x higher than in CA plants but only 0.7x as high as in V-CA, whereas for *CR7* the expression level was similar in CA, V-CA, and RA. The two F_2_-populations had similar gene expression patterns, except for *CR7*, which was significantly different, with VRmax having almost twice as high expression of *CR7* as VRmin in the CA and RA treatments (**Figure 5**). There were no significant differences in gene expression between the two phenotypic classes of VRmin, but there were some differences between the genotypic classes based on the haplotype defined by the *b*-allele of *VRN1*. The *b*-haplotype was not significantly associated with *VRN1* expression level, but it was associated with lower expression of *COR14B* in the V-CA and DA treatments and of *CBF6* and *LOS2* in the DA treatment (**Figure 6**). There were also some weak, but significant, correlations between expression levels of different genes, and between expression of specific genes and PHS and LT50 in VRmin (**Table 4**). The strongest correlations (positive) were found between expression of *VRN1, CBF6, COR14B*, and *LOS2* in the CA, DA, and RA temperature treatments, and there were also significant positive correlations between expression of these genes and *IRI1*.

**Table 4:** Correlation coefficients (R) between variables measured for 21 genotypes in the *Festuca pratensis* VRmin population given four different temperature treatments (see **Figure 1**).

Variables/temperature treatments		PHS	DTH	LT50	*VRN1*	*COR14B*	*CR7*	*CBF6*	*IRI1*
DTH	CA	–0.89^∗∗∗^							
	V-CA	–0.75^∗∗∗^							
	DA	–0.49^∗^							
	RA	NS							
LT50	CA	NS	NS						
	V-CA	NS	NS						
	DA	NS	NS						
	RA	NS	NS						
*VRN1*	CA	NS	NS	NS					
	V-CA	NS	NS	NS					
	DA	NS	NS	NS					
	RA	NS	NS	NS					
*COR14B*	CA	NS	NS	NS	0.63^∗∗^				
	V-CA	NS	NS	NS	NS				
	DA	NS	NS	–0.47^∗^	0.55^∗∗^				
	RA	NS	NS	NS	0.59^∗∗^				
*CR7*	CA	NS	NS	NS	NS	NS			
	V-CA	NS	NS	NS	NS	NS			
	DA	NS	NS	NS	NS	NS			
	RA	NS	NS	–0.46^∗^	NS	NS			
*CBF6*	CA	NS	NS	NS	0.58^∗∗^	0.75^∗∗∗^	NS		
	V-CA	NS	NS	NS	NS	NS	NS		
	DA	NS	NS	NS	NS	NS	NS		
	RA	NS	NS	NS	0.66^∗∗^	0.61^∗^	NS		
*IRI1*	CA	NS	NS	NS	NS	0.59^∗∗^	NS	NS	
	V-CA	NS	NS	NS	0.49^∗^	NS	NS	NS	
	DA	NS	NS	NS	NS	NS	NS	NS	
	RA	0.56^∗^	NS	NS	0.55^∗^	NS	NS	NS	
*LOS2*	CA	NS	NS	NS	0.58^∗∗^	0.51^∗^	NS	0.48^∗^	NS
	V-CA	NS	NS	NS	0.43^∗^	NS	NS	NS	0.49^∗^
	DA	NS	NS	NS	NS	0.62^∗∗^	NS	0.44^∗^	NS
	RA	NS	NS	NS	NS	NS	NS	NS	0.55^∗^

*PHS, percent of heading shoots; DTH, days to heading; LT50, freezing temperature at which 50% of the plants are estimated to die. Remaining variables are expression levels of various genes in shoot basis tissue. ^∗∗∗^*P* < 0.001; ^∗∗^0.01 < *P* < 0.001; ^∗^0.01 < *P* < 0.05; NS, 0.05 < *P*. Note that a negative correlation with LT50 indicates a positive correlation with freezing tolerance. Expression of *MADS3* was not significantly correlated with any of the other variables (not shown).*

## Discussion

### Flowering Response

The different vernalization response in the two F_2_-populations, and the segregation within VRmin for the ability to head without vernalization reported previously (Ergon et al., 2013), was confirmed in the experiment reported here. The ability to head without vernalization and the heading responses after 9 weeks of vernalization were not associated, and hence appear to be controlled by different genetic factors. As expected, the two F_2_ populations differed in the level of vernalization saturation after 9 weeks of cold treatment (V-CA). Although the *b*-haplotype was associated with the ability to flower without vernalization in the F_1_ generation (Ergon et al., 2006), it was not associated with this trait in the VRmin F_2_ population, which segregated for this trait, and after vernalization it was instead associated with later heading. This difference between generations could be due to epistatic effects being masked in the F_1_ generation or due to an effect of the other maternal haplotype, which could not be identified with the CAPS-marker.

Interestingly, plants from the DA treatment headed 10 days earlier than plants exposed to the V-CA treatment only (counted from the time of transfer to the greenhouse after the temperature treatment was ended). This shows that the reproductive development progressed at the same, or at a slightly faster, rate during DA (12°C, 8 h photoperiod), as compared to greenhouse conditions (∼18°C, 16 h photoperiod), in spite of a lower temperature. Short photoperiods stimulate induction of flowering and to some extent replace vernalization in perennial grasses, while long photoperiods accelerate flowering in vernalized plants and in plants without a vernalization requirement (Heide, 1988, 1994). The effect of photoperiod during the different stages of meristem and inflorescence development has not been described in detail in grasses; the effect may vary between species and genotypes. In the partially vernalized *F. pratensis* in our experiment, short photoperiods appeared to be more efficient in promoting floral development than long photoperiods as it was able to compensate for the difference in temperature.

### Effects of Temperature Treatments on Gene Expression

The ability of some VRmin genotypes to head after only 2 weeks of cold (CA) was not significantly associated with higher *VRN1* or *MADS3* expression levels at the shoot basis in this temperature treatment. In previous experiments (Ergon et al., 2013), the ability to head without vernalization was associated with a higher expression of these two genes, particularly *MADS3*, in non-vernalized plants. In those experiments non-vernalized plants were grown under an 18 h photoperiod in the greenhouse prior to sampling and not 12 h followed by 8 h at 2°C as in the experiment reported here. The expression of *VRN1* and *MADS3* in non-vernalized plants of the VRmin- phenotypic class may depend on long days. There is little information available on expression patterns of *MADS3* in other species. *VRN1* is known to be induced or enhanced by long days in leaves and shoot apices of temperate grasses if certain conditions are met, i.e., after vernalization (Sasani et al., 2009, *Hordeum vulgare*), after a long period of short days (Dubcovsky et al., 2006, *Triticum aestivum*), and in genotypes that are not restricted by a requirement for vernalization and/or a short day period in order to flower (Gocal et al., 2001, *Lolium temulentum*). It is not clear whether *VRN1* expression is actually required for transition to reproductive development in these cases. Instead, it may be upregulated in the apex after transition, where it appears to have a role in the development of the inflorescence and flowers (Gocal et al., 2001; Preston and Kellogg, 2007). This could also be the case for *MADS3*.

The genes that we studied differed in the way their expression was affected by the prolonged cold treatment. There were no significant differences between the CA and V-CA treatments in the expression of *CBF6, COR14B*, and *CR7*, but *VRN1, MADS3, LOS2*, and *IRI1* were all present at higher transcript levels after the V-CA than after the CA treatment. The level of *VRN1* expression increases during prolonged cold in winter cereals with a vernalization response (reviewed by Trevaskis, 2010), and this appears to also be the case in perennial grasses, such as *L. perenne* (Petersen et al., 2004) and *Phleum pratense* (Seppänen et al., 2010). *MADS3* was more strongly expressed in *L. perenne* after 12 weeks at 5°C and 8 h photoperiod than after 6 weeks (Petersen et al., 2004). *LOS2* tended to have a higher expression in *F. pratensis* after 18 and 21 days at 4/2°C (day/night) and 10 h photoperiod than after 1 day (Jurczyk et al., 2013a,b). *IRI*-genes have been shown to be cold induced in leaves of *L. perenne* (Zhang et al., 2010), *Brachypodium distachyon* (Li et al., 2012; Colton-Gagnon et al., 2014) and *Deschampsia antarctica* (Chew et al., 2012). In these studies, expression was only tested for up to 1–2 weeks of cold exposure, except for Colton-Gagnon et al. (2014), who found a decline in expression from 1 to 2 weeks up to 5 weeks.

*CBF6* did not change significantly in expression levels from 2 weeks (CA) to 9 weeks (V-CA) of cold, it was not down-regulated by deacclimation, and it was expressed at a higher level after reacclimation than before deacclimation. This is in contrast to many *CBF*-genes, which are induced rapidly by cold, and then revert to a basic level. *CBF* genes vary in their expression patterns, however. Among the wheat and barley *CBF* genes (Skinner et al., 2005), *FpCBF6* has the highest identity with *HvCBF6* and *TaCBF6. HvCBF6* was found to have a delayed cold response with a peak in expression at 24 h after transfer to cold (2°C, 16 h photoperiod), and then maintained at that level (Skinner et al., 2005).

Across all genotypes *COR14B* and *CR7* expression was also maintained during prolonged cold in our experiment, without any significant change in expression levels between plants exposed to 7 weeks of vernalization temperatures +2 weeks of cold (V-CA) compared with those exposed only to 2 weeks of cold (CA). Studies of cereals have reported both up- and downregulation of *COR14B* in response to prolonged cold. The outcome appears to be related to photoperiod. Dhillon et al. (2010) found that in *Triticum monococcum, COR14B* was down-regulated during prolonged cold (lower expression level after six compared to 2 weeks), and more so under long days (16 h photoperiod) than under short days (8 h photoperiod). In line with this, in several studies of wheat a decline in the expression of *COR14B* was found during prolonged cold in long days (16 h photoperiod, Ganeshan et al., 2008; Laudencia-Chingcuanco et al., 2011). Barley seedlings germinated in darkness, however, had higher expression of *COR14B* after exposure to 7 weeks of cold than after only 4 days of cold (Greenup et al., 2011). Similarly, Gana et al. (1997) found an increase in the expression of *CR7* in crown tissue of wheat seedlings exposed to cold in darkness for up to 4 weeks. *COR14B* is known to be regulated by light-dependent factors (Crosatti et al., 1995, 1999), but this has not been described for *CR7*. The long day-induced gene *FT1/VRN3* (Turner et al., 2005) may play a role in this as the locus harboring this gene is found to affect *COR14B* expression in barley (Cuesta-Marcos et al., 2015).

We found that all the studied genes except *CBF6* were significantly downregulated in the shoot basis after 1 week of deacclimation at 12 h photoperiod. *VRN1* was also down-regulated in above-ground parts of *B. distachyon* when vernalized plants were placed at growth temperatures (16 h photoperiod, Colton-Gagnon et al., 2014). Similarly, Greenup et al. (2011) found a down-regulation of *VRN1* in etiolated and vernalized barley seedlings when exposed to growth temperatures. The expression in deacclimated plants in both these studies were still higher than that in non-vernalized plants, and when etiolated and vernalized barley seedlings were placed in the greenhouse with a 16 h photoperiod, *VRN1* expression in leaf blades remained high (Greenup et al., 2011). Sasani et al. (2009) had previously found that this expression in leaves was lower under short days (8 h photoperiod) than under long days (16 h photoperiod), while the expression in the shoot apices was not sensitive to photoperiod. Taken together, these and our results suggest that *VRN1* is downregulated in shoot apices by deacclimation, but not to the level of non-vernalized plants, and that in leaf blades, but not apices/stem bases, the down-regulation is limited to short day conditions. Inclusion of other tissues than leaf blades, or species differences, may explain the down-regulation observed in shoots of *B. distachyon* by Colton-Gagnon et al. (2014).

It was only *VRN1* and *CR7* that were significantly upregulated by reacclimation relative to the deacclimated plants. Two weeks of reacclimation after deacclimation resulted in different expression levels than the two first weeks of CA for *CBF6* (discussed above), *VRN1* and *COR14B*. *VRN1* expression was higher after RA than after CA, while the opposite occurred for *COR14B*. *CR7, LOS2*, and *IRI1* were expressed at similar levels after CA and RA. Thus, it appears that *VRN1* expression is somehow primed after prolonged cold; this may be related to changes in chromatin structure showed to occur at the *VRN1* locus (Oliver et al., 2009, 2013). The lower expression of *COR14B*, but not *CR7*, after RA compared with CA, is interesting. Laudencia-Chingcuanco et al. (2011) studied the effect of the *VRN1* locus on the expression of *COR14B* and *BLT14.1* in wheat during prolonged cold treatment under 16 h photoperiods (*BLT14.1* is the wheat/barley *CR7/BLT*-gene with the highest similarity to *FpCR7*). Expression of both genes was affected by the *VRN1* locus; their expression levels increased or was maintained for a longer time when the spring allele of *VRN1* was absent and the expression of *VRN1* was delayed. Expression of *COR14B*, however, was more strongly reduced after 10 weeks of cold than *BLT14.1* (*CR7*). Our results are in agreement with this, and shows that vernalization may also have a down-regulating effect on *COR14B*, but not *CR7*, under short photoperiods.

### Freezing Tolerance as Affected by Vernalization and the *VRN1* Chromosomal Region

The two phenotypic classes of VRmin differed in freezing tolerance in the CA treatment, the only treatment for which they also displayed different heading phenotypes. Here, the tendency to head was associated with better freezing tolerance. This effect may be attributable to genetic linkage. The genetic control of the ability to head without vernalization in the F_1_ generation of this plant material is mainly controlled by loci on chromosome 4F, of which some, but not the strongest ones, are closely linked to *VRN1* (Ergon et al., 2006). There is also a QTL for freezing tolerance after 2 weeks of CA located close to *VRN1* (Alm et al., 2011), which may account for the difference in freezing tolerance between the two phenotypic classes. Indeed, a re-examination of the marker data of Alm et al. (2003) showed that, in the F_1_ population, the maternal marker haplotype of the *VRN1*-region associated with the ability to head without vernalization was also the one that was associated with better freezing tolerance after 2 weeks of CA (the paternal haplotypes did not have different effects on these traits in F_1_). Šimkūnas et al. (2013) also found a positive correlation among Festulolium cultivars between heading prior to vernalization and survival in the field during the following winter. They speculated that this might be due to more young tillers in the plants heading prior to vernalization, but had no data supporting this. An alternative explanation could be a linkage between alleles conferring the ability to head without vernalization and freezing tolerance. The effect of the *b*-haplotype on freezing tolerance observed in the F_1_ generation was retained in VRmin, and appeared to be recessive. No effect of the *b*-haplotype on freezing tolerance was seen after prolonged cold or after a deacclimation–reacclimation cycle, thus it appears to be involved in the relatively early stages of CA. The *b*-haplotype was also associated with later and less heading after the vernalization treatment, i.e., a lower responsiveness to vernalization, also in a recessive manner. The ability to flower without vernalization on one hand, and vernalization response on the other hand, may be controlled by different genetic factors. Indeed, in our previous QTL analysis using the F_1_ generation, the trait “vernalization requirement” included both these traits but was dominated by the variation in the ability to flower without vernalization, and the QTLs of largest magnitude were located 10 cM proximal to *VRN1* (Ergon et al., 2006). In cereals, a QTL conferring freezing tolerance *(Fr-1)* is closely linked to *VRN1* (Sutka and Snape, 1989), and *VRN1* alleles conferring a vernalization requirement/slower vernalization response in cereals is associated with better freezing tolerance, similarly to the *b*-haplotype in our material. Based on phenotypic characterization (freezing tolerance, gene expression) of *Triticum monococcum* mutants where a small region encompassing *VRN1* and a few more genes had been deleted, it was suggested that *VRN1* is actually responsible for both traits (Dhillon et al., 2010). We found that freezing tolerance increased substantially by the vernalization treatment (7 weeks at 6°C) prior to the 2 weeks of CA at 2°C. In the V-CA treatment there was no difference in freezing tolerance between the two F_2_ populations or between the phenotypic classes of VRmin. Reacclimation after deacclimation was efficient, but VRmin had a significantly lower freezing tolerance after RA than after CA, while VRmax did not, indicating some effect of the degree of vernalization on the de-acclimation/re-acclimation process, even under short photoperiods. The *VRN1 b*-haplotype was associated with better freezing tolerance in the CA treatment and less restoration of freezing tolerance in the RA treatment. The *b*-haplotype was also associated with later heading and a lower percent of heading shoots per plant in the vernalized treatments, and with a lower level of *CBF6, COR14B*, and *LOS2* expression in vernalized and/or deacclimated treatments. This suggests that the *VRN1*-region somehow regulates the expression of these genes in vernalized plants or during deacclimation, and that less saturation of the vernalization requirement is associated with lower expression of cold-induced genes. In cereals, the opposite relationship has been found; vernalization saturation is associated with a down-regulation of cold-induced genes through mechanisms controlled by the *VRN1*-region (Fowler et al., 1996; Limin and Fowler, 2006; Laudencia-Chingcuanco et al., 2011). However, as shown by Dhillon et al. (2010), the effect that the *VRN1* locus has on down-regulation of cold-induced genes, when the vernalization requirement is saturated, is dependent on long photoperiods. The short photoperiod used in our cold treatments, representing Norwegian mid-winter/early spring conditions, is a possible explanation for why we did not observe a similar association between vernalization saturation or vernalization requirement and down-regulation of cold-induced genes. We observed some correlation between *VRN1* expression and expression of *CBF6, COR14B, LOS2*, and *IRI1*, and these correlations were always positive. Oliver et al. (2013) observed similar expression patterns of *VRN1* and *COR14B* in barley seedlings in the dark, and suggested that these genes may be regulated by similar mechanisms in early CA, possibly through the action of CBF transcription factors. Long photoperiods appear to disrupt this co-regulation, possibly through an interaction with *FT1/VRN3*. *MADS3* and *CR7* appeared to be regulated by other mechanisms. While expression of *COR14B* could explain some of the variation in freezing tolerance in deacclimated plants, *CR7* was, in addition to *VRN1*, the only gene that was significantly upregulated by reacclimation and that could explain some of the variation in freezing tolerance after reacclimation. This suggests that *CR7* may have a particular role after a cycle of deacclimation and reacclimation.

## Conclusion

**FIGURE 7:**
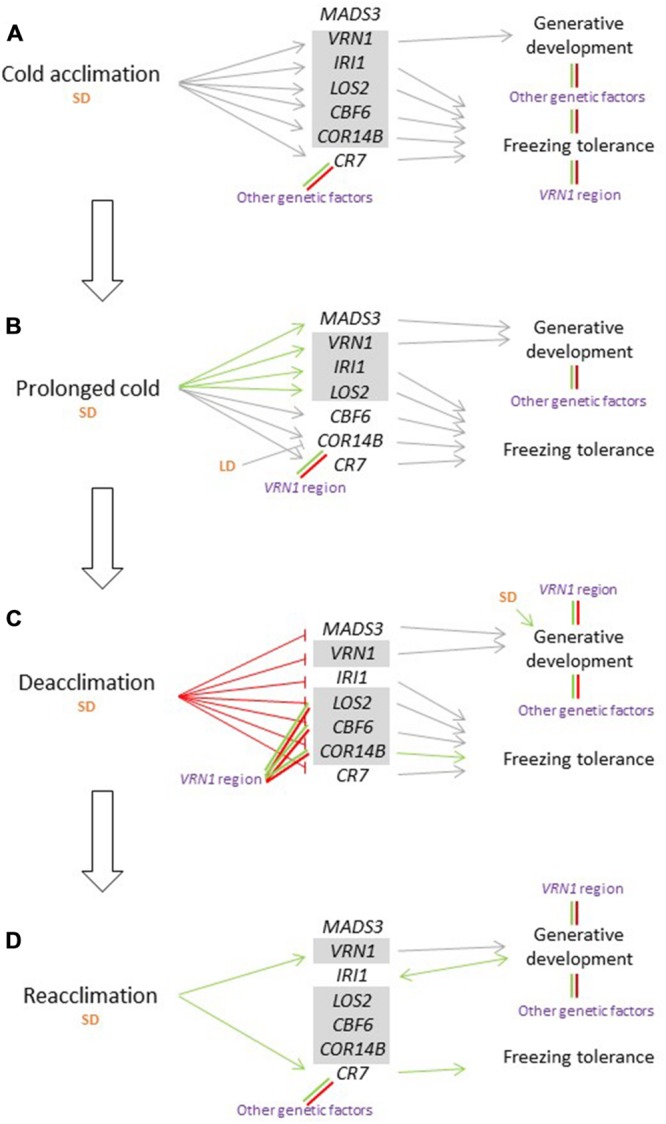
**Model for the effects of different temperatures, in sequential order from cold acclimation **(A)** and prolonged cold **(B)** to deacclimation **(C)** and reacclimation **(D)**, all at short day conditions (SD), on gene expression, generative development, and freezing tolerance in *Festuca pratensis*.** A pointed arrow indicates a positive, and a blunt arrow a negative, effect on, or association with, a process. Green and red arrows are supported by the data presented here, gray arrows by the discussed literature (including other temperate grass species). Combined green and red lines indicate segregating effects observed here. Gray boxes indicate sets of co-regulated genes. LD, long days.

Some genotypes of *F. pratensis* are able to head to a limited extent without vernalization. This ability appears to be controlled by other genetic factors than the *VRN1*-region and is not associated with the responsiveness to vernalization or timing of heading. Timing of heading is associated with the *VRN1*-region but also with other genetic factors. Under short day conditions *VRN1, CBF6, COR14B, LOS2*, and *IRI1* appear to be largely co-regulated, while *CR7* and *MADS3* are regulated by other mechanisms. Our results indicate that the relationship between vernalization and freezing tolerance in *F. pratensis* is complex. After 2 weeks of CA (**Figure 7A**), the genotypes that are able to head to a limited extent also has a better freezing tolerance, and the *VRN1*-region also has some effect on freezing tolerance. *CR7* is more strongly expressed in genotypes with a higher vernalization requirement, but this does not result in better freezing tolerance at this stage. During prolonged cold (**Figure 7B**), *VRN1, LOS2, IRI1*, and *MADS3* continue to be upregulated, while the expression of *CBF6, COR14B*, and *CR7* are maintained at a constant level. The *VRN1*-region has some effect on the expression of *COR14B*. During deacclimation (**Figure 7C**), *VRN1, LOS2, IRI1, MADS3, COR14B*, and *CR7* are down-regulated, and the expression of *LOS2, CBF6*, and *COR14B* is affected by the *VRN1*-region. At this stage expression of *COR14B* is associated with better freezing tolerance. During reacclimation (**Figure 7D**), *VRN1* and *CR7* are upregulated, while the expression of other cold-induced genes remains relatively stable. At this stage expression of *CR7*, which is associated with a lower vernalization requirement, is associated with better freezing tolerance.

Our results indicate that in *F. pratensis*, some genotypes can more easily lose some freezing tolerance after a deacclimation–reacclimation cycle, even under short photoperiods. The effect appears to be limited, but may increase significantly as photoperiods rapidly become longer than 12 h after the spring equinox. The role of the photoperiod in deacclimation and reacclimation processes in *F. pratensis* and other perennial grasses cultivated or growing naturally in the north, where photoperiods become long while there is still a risk of freezing, deserves further investigation.

## Author Contributions

ÅE, MH, and OR designed the study. ÅE conducted the experiment, analyzed the data, and wrote the manuscript. TM performed the qRT-PCR analysis. All authors read, revised, and approved the manuscript.

## Conflict of Interest Statement

The authors declare that the research was conducted in the absence of any commercial or financial relationships that could be construed as a potential conflict of interest.

## Abbreviations

CAPS: cleaved amplified polymorphic sequence
DTH: days to heading
PAR: photosynthetically active radiation
PHP: percent heading plants
PHS: percent of heading shoots per plant
qRT-PCR: quantitative reverse transcription-polymorphic chain reaction

## References

[B1] Alm V. Busso C. S. Ergon Å Rudi H. Larsen A. Humphreys M. W. (2011). QTL analyses and comparative mapping of frost tolerance, winter survival and drought tolerance in meadow fescue (*Festuca pratensis* Huds.). *Theor. Appl. Genet.* 123 369–382. 10.1007/s00122-011-1590-z 21505831

[B2] Alm V. Fang C. Busso C. S. Devos K. M. Vollan K. Grieg Z. (2003). A linkage map of meadow fescue (*Festuca pratensis* Huds.) and comparative mapping with other *Poaceae* species. *Theor. Appl. Genet.* 108 25–40. 10.1007/s00122-003-1399-5 12923626

[B3] Bokhorst S. F. Bjerke J. W. Tømmervik H. Callaghan T. V. Phoenix G. K. (2009). Winter warming events damage sub-Arctic vegetation: consistent evidence from an experimental manipulation and a natural event. *J. Ecol.* 97 1408–1415. 10.1111/j.1365-2745.2009.01554.x

[B4] Chew O. Lelean S. John U. P. Spangenberg G. C. (2012). Cold acclimation induces rapid and dynamic changes in freeze tolerance mechanisms in the cryophile *Deschampsia antarctica* E. Desv. *Plant Cell Environ.* 35 829–837. 10.1111/j.1365-3040.2011.02456.x 22070607

[B5] Colton-Gagnon K. Ali-Benail M. A. Mayer B. F. Dionne R. Bertrand A. Carmo S. D. (2014). Comparative analysis of the cold acclimation and freezing tolerance capacities of seven diploid *Brachypodium distachyon* accessions. *Ann. Bot.* 113 681–693. 10.1093/aob/mct283 24323247 PMC3936580

[B6] Crosatti C. Marè C. Mazzucotelli E. Belloni S. Barilli S. Bassi R. (2003). Genetic analysis of the expression of the cold regulated gene cor14b: a way toward the identification of components of the cold response signal transduction in Triticeae. *Can. J. Bot.* 81 1162–1167. 10.1139/b03-114

[B7] Crosatti C. Polverino de Laureto P. Bassi R. Cattivelli L. (1999). The interaction between cold and light controls the expression of the cold-regulated barley gene cor14b and the accumulation of the corresponding protein. *Plant Physiol.* 119 671–680. 10.1104/pp.119.2.671 9952464 PMC32145

[B8] Crosatti C. Soncini C. Stanca A. M. Cattivelli L. (1995). The accumulation of a cold- regulated chloroplastic protein is light-dependent. *Planta* 196 458–463. 10.1007/BF00203644 7647681

[B9] Cuesta-Marcos A. Muñoz-Amatriaín M. Filichkin T. Karsai I. Trevaskis B. Yasuda S. (2015). The relationships between development and low temperature tolerance in barley near isogenic lines differing for flowering behavior. *Plant Cell Physiol.* 56 2312–2324. 10.1093/pcp/pcv147 26443377

[B10] Dal Bosco C. Busconi M. Govoni C. Baldi P. Stanca A. M. Crosatti C. (2003). cor gene expression in barley mutants affected in chloroplast development and photosynthetic electron transport. *Plant Physiol.* 131 793–802. 10.1104/pp.014530 12586903 PMC166855

[B11] Dhillon T. Pearce S. P. Stockinger E. J. Distelfeld A. Li C. Knox A. K. (2010). Regulation of freezing tolerance and flowering in temperate cereals: the VRN-1 connection. *Plant Physiol.* 153 1846–1858. 10.1104/pp.110.159079 20571115 PMC2923912

[B12] Dubcovsky J. Loukoianov A. Fu D. Valarik M. Sanchez A. Yan L. (2006). Effect of photoperiod on the regulation of wheat vernalization genes VRN1 and VRN2. *Plant Mol. Biol.* 60 469–480. 10.1007/s11103-005-4814-2 16525885 PMC4739792

[B13] Ergon Å Fang C. Jørgensen Ø Aamlid T. S. Rognli O. A. (2006). Quantitative trait loci controlling vernalization requirement, heading time and number of panicles in meadow fescue (*Festuca pratensis* Huds.). *Theor. Appl. Genet.* 112 232–242. 10.1007/s00122-005-0115-z 16235049

[B14] Ergon Å Hamland H. Rognli O. A. (2013). Differential expression of VRN1 and other MADS-box genes in *Festuca pratensis* selections with different vernalization requirements. *Biol. Plant.* 57 245–254. 10.1007/s10535-012-0283-z

[B15] Espevig T. Höglind M. Aamlid T. S. (2014). Dehardening resistance of six turfgrasses used on golf greens. *Environ. Exp. Bot.* 106 182–188. 10.1016/j.envexpbot.2014.02.006

[B16] Fjellheim S. Boden S. Trevaskis B. (2014). The role of seasonal flowering responses in adaptation of grasses to temperate climates. *Front. Plant Sci.* 5:431 10.3389/fpls.2014.00431 PMC4148898 25221560

[B17] Fowler D. B. Chauvin L. P. Limin A. E. Sarhan F. (1996). The regulatory role of vernalization in the expression of low-temperature-induced genes in wheat and rye. *Theor. Appl. Genet.* 93 554–559. 10.1007/BF00417947 24162347

[B18] Galiba G. Vágújfalvi A. Li C. Soltesz A. Dubcovsky J. (2009). Regulatory genes involved in the determination of frost tolerance in temperate cereals. *Plant Sci.* 176 12–19. 10.1016/j.plantsci.2008.09.016

[B19] Gana J. A. Sutton F. Kenefick D. G. (1997). cDNA structure and expression patterns of a low-temperature-specific wheat gene tacr7. *Plant Mol. Biol.* 34 643–650. 10.1023/A:1005852703506 9247545

[B20] Ganeshan S. Vitamvas P. Fowler D. B. Chibbar R. N. (2008). Quantitative expression analysis of selected COR genes reveals their differential expression in leaf and crown tissues of wheat (*Triticum aestivum* L.) during an extended low temperature acclimation regimen. *J. Exp. Bot.* 59 2393–2402. 10.1093/jxb/ern112 18508811 PMC2423658

[B21] Gay A. P. Eagles C. F. (1991). Quantitative analysis of cold hardening and dehardening in Lolium. *Ann. Bot.* 67 339–345.

[B22] Gocal G. F. W. King R. W. Blundell C. A. Schwartz O. M. Andersen C. H. Weigel D. (2001). Evolution of floral meristem identity genes. Analysis of Lolium temulentum genes related to APETALA1 and LEAFY of *Arabidopsis*. *Plant Physiol.* 125 1788–1801. 10.1104/pp.125.4.1788 11299359 PMC88835

[B23] Greenup A. G. Sasani S. Oliver S. N. Walford S. A. Millar A. A. Trevaskis B. (2011). Transcriptome analysis of the vernalization response in barley (*Hordeum vulgare*) seedlings. *PLoS ONE* 6:e17900 10.1371/journal.pone.0017900 PMC3052371 21408015

[B24] Gu L. Hanson P. J. Post W. M. Kaiser D. P. Yang B. Nemani R. (2008). The 2007 eastern US spring freeze: increased cold damage in awarming world? *Bioscience* 58 253–262. 10.1641/B580311

[B25] Heide O. M. (1988). Flowering requirements of Scandinavian *Festuca pratensis*. *Physiol. Plant.* 74 487–492. 10.1111/j.1399-3054.1988.tb02007.x

[B26] Heide O. M. (1994). Control of flowering and reproduction in temperate grasses. *New Phytol.* 128 347–362. 10.1111/j.1469-8137.1994.tb04019.x 33874362

[B27] Hoffmann L. DaCosta M. Ebdon J. S. (2014). Examination of cold deacclimation sensitivity of annual bluegrass and creeping bentgrass. *Crop Sci.* 54 413–420. 10.2135/cropsci2013.05.0329

[B28] Jensen L. B. Andersen J. R. Frei U. Xing Y. Taylor C. Holm P. B. (2005). QTL mapping of vernalization response in perennial ryegrass (*Lolium perenne* L.) reveals co-location with an orthologue of wheat VRN1. *Theor. Appl. Genet.* 110 527–536. 10.1007/s00122-004-1865-8 15619078

[B29] Johansson C. Pohjola V. A. Jonasson C. Callaghan T. V. (2011). Multi-decadal changes in snow characteristics in sub-Arctic Sweden. *Ambio* 40 566–574. 10.1007/s13280-011-0164-2 21954720 PMC3357863

[B30] Jørgensen M. Østrem L. Höglind M. (2010). De-hardening in contrasting cultivars of timothy and perennial ryegrass during winter and spring. *Grass For. Sci.* 65 38–48. 10.1111/j.1365-2494.2009.00718.x

[B31] Jurczyk B. Krespi T. Kosmala A. Rapacz R. (2013b). Different mechanisms trigger an increase in freezing tolerance in *Festuca pratensis* exposed to flooding stress. *Environ. Exp. Bot.* 93 45–54. 10.1016/j.envexpbot.2013.06.003

[B32] Jurczyk B. Rapacz M. Budzisz K. Barcik W. Sasal M. (2012). The effects of cold, light and time of day during low-temperature shift on the expression of CBF6, FpCor14b and LOS2 in *Festuca pratensis*. *Plant Sci.* 183 143–148. 10.1016/j.plantsci.2011.08.004 22195587

[B33] Jurczyk B. Rapacz R. Krespi T. (2013a). Short-term growth of meadow fescue with atmospheric decreases freezing tolerance, modifies photosynthetic apparatus performance and changes the expression of some genes during cold acclimation. *Acta Physiol. Plant.* 35 1543–1554. 10.1007/s11738-012-1196-3

[B34] Kalberer S. R. Wisniewski M. Arora R. (2006). Deacclimation and reacclimation of cold-hardy plants: current understanding and emerging concepts. *Plant Sci.* 171 3–16. 10.1016/j.plantsci.2006.02.013

[B35] Laudencia-Chingcuanco D. Ganeshan S. You F. Fowler B. Chibbar R. Anderson O. (2011). Genome-wide gene expression analysis supports a developmental model of low temperature tolerance gene regulation in wheat (*Triticum aestivum* L.). *BMC Genomics* 12:99 10.1186/1471-2164-12-299 PMC3141665 21649926

[B36] Lee H. Guo Y. Ohta M. Xiong L. Stevenson B. Zhu J.-K. (2002). LOS2, a genetic locus required for cold-responsive gene transcription encondes a bi-functional enolase. *EMBO J.* 21 2692–2702. 10.1093/emboj/21.11.2692 12032082 PMC126021

[B37] Li C. Rudi H. Stockinger E. J. Cheng H. Cao M. Fox S. E. (2012). Comparative analysis reveal potential uses of Brachypodium distachyon as a model for cold stress responses in temperate grasses. *BMC Plant Biol.* 12:65 10.1186/1471-2229-12-65 PMC3487962 22569006

[B38] Limin A. E. Fowler D. B. (2006). Low-temperature tolerance and genetic potential in wheat (*Triticum aestivum* L.): response to photoperiod, vernalization, and plant development. *Planta* 224 360–366. 10.1007/s00425-006-0219-y 16440213

[B39] Mahfoozi S. Limin A. E. Ahakpaz F. Fowler D. B. (2006). Phenological development and expression of freezing resistance in spring and winter wheat under field conditions in northwest Iran. *Field Crops Res.* 97 182–187. 10.1016/j.fcr.2005.09.012

[B40] Mahfoozi S. Limin A. E. Ahakpaz F. Roustaii M. Ketata H. Fowler D. B. (2005). Regulation of low-temperature tolerance in barley under field conditions in northwest Iran. *Can. J. Plant Sci.* 85 587–592. 10.4141/P04-191

[B41] Mahfoozi S. Limin A. E. Fowler D. B. (2001a). Influence of vernalization and photoperiod responses on cold hardiness in winter cereals. *Crop Sci.* 41 1006–1011. 10.2135/cropsci2001.4141006x

[B42] Mahfoozi S. Limin A. E. Fowler D. B. (2001b). Developmental regulation of low-temperature tolerance in winter wheat. *Ann. Bot.* 87 751–757. 10.1006/anbo.2001.1403

[B43] Oliver S. N. Deng W. Casao M. C. Trevaskis B. (2013). Low temperatures induce rapid changes in chromatin state and transcript levels of the cereal VERNALIZATION1 gene. *J. Exp. Bot.* 64 2413–2422. 10.1093/jxb/ert095 23580755 PMC3654426

[B44] Oliver S. N. Finnegan E. J. Dennis E. S. Peacock W. J. Trevaskis B. (2009). Vernalization-induced flowering in cereals is associated with changes in histone methylation at the VERNALIZATION1 gene. *Proc. Natl. Acad. Sci. U.S.A.* 106 8386–8391. 10.1073/pnas.0903566106 19416817 PMC2677093

[B45] Pearce R. S. Houlston C. E. Atherton K. M. Rixon J. E. Harrison P. Hughes M. A. (1998). Localization of expression of three cold-induced genes, blt101, blt4.9, and blt14, in different tissues of the crown and developing leaves of cold-acclimated cultivated barley. *Plant Physiol.* 117 787–795. 10.1104/pp.117.3.787 9662521 PMC34933

[B46] Penfield S. (2008). Temperature perception and signal transduction in plants. *New Phytol.* 179 615–628. 10.1111/j.1469-8137.2008.02478.x 18466219

[B47] Petersen K. Didion T. Andersen C. H. Nielsen K. K. (2004). MADS-box genes from perennial ryegrass differentially expressed during transition from vegetative to reproductive growth. *J. Plant Physiol.* 161 439–447. 10.1078/0176-1617-01212 15128031

[B48] Phillips J. R. Dunn M. A. Hughes M. A. (1997). mRNA stability and localisation of the low-temperature-responsive barley gene family blt14. *Plant Mol. Biol.* 33 1013–1023. 10.1023/A:1005717613224 9154983

[B49] Preston J. Kellogg E. A. (2008). Discrete developmental roles for temperate cereal grass VERNALIZATION1/FRUITFUL-like genes in flowering competency and the transition to flowering. *Plant Physiol.* 146 265–276. 10.1104/pp.107.109561 18024551 PMC2230560

[B50] Preston J. C. Kellogg E. A. (2007). Conservation and divergence of APETALA1/FRUITFULL-like gene function in grasses: evidence from gene expression analyses. *Plant J.* 52 69–81. 10.1111/j.1365-313X.2007.03209.x 17666026

[B51] Rapacz M. Ergon Å Höglind M. Jørgensen M. Jurczyk B. Østrem L. (2014). Overwintering of herbaceous plants in a changing climate. Still more questions than answers. *Plant Sci.* 225 34–44. 10.1016/j.plantsci.2014.05.009 25017157

[B52] Rudi H. Sandve S. R. Opseth L. M. Larsen A. Rognli O. A. (2011). Identification of candidate genes important for frost tolerance in *Festuca pratensis* Huds. by transcriptional profiling. *Plant Sci.* 180 78–85. 10.1016/j.plantsci.2010.07.014 21421350

[B53] Sandve S. R. Kosmala A. Rudi H. Fjellheim S. Rapacz M. Yamada T. (2011). Molecular mechanisms underlying frost tolerance in perennial grasses adapted to cold climates. *Plant Sci.* 180 69–77. 10.1016/j.plantsci.2010.07.011 21421349

[B54] Sandve S. R. Rudi H. Asp T. Rognli O. A. (2008). Tracking the evolution of a cold stress associated gene family in cold tolerant grasses. *BMC Evol. Biol.* 8:245 10.1186/1471-2148-8-245 PMC2542378 18775065

[B55] Sasani S. Hemming M. N. Oliver S. N. Greenup A. Tavakkol-Afshari R. Mahfoozi S. (2009). The influence of vernalization and daylength on expression of flowering-time genes in the shoot apex and leaves of barley (*Hordeum vulgare*). *J. Exp. Bot.* 60 2169–2178. 10.1093/jxb/erp098 19357429 PMC2682508

[B56] Schmitz J. Franzen R. Ngyuen T. H. Garcia-Maroto F. Pozzi C. Salamini F. (2000). Cloning, mapping and expression analysis of barley MADS-box genes. *Plant Mol. Biol.* 42 899–913. 10.1023/A:1006425619953 10890536

[B57] Seppänen M. Pakarinen K. Jokela V. Andersen J. R. Fiil A. Santanen A. (2010). Vernalization response of *Phleum* pratense and its relationships to stem lignification and floral transition. *Ann. Bot.* 106 697–707. 10.1093/aob/mcq174 20798263 PMC2958789

[B58] Shabbar A. Bonsal B. (2003). An assessment of changes in winter cold and warm spells over Canada. *Nat. Haz.* 29 173–188. 10.1023/A:1023639209987

[B59] Šimkūnas A. Valaŝinaite S. Denisov V. Salytė A. (2013). Systemic view on heading and overwintering: are they always opposed? *J. Agron. Crop Sci* 199 460–465. 10.1111/jac.12029

[B60] Skinner J. S. von Zitzewitz J. Szücs P. Marquez-Cedillo L. Filichkin T. Amundsen K. (2005). Structural, functional, and phylogenetic characterization of a large CBF gene family in barley. *Plant Mol. Biol.* 59 533–551. 10.1007/s11103-005-2498-2 16244905

[B61] Sutka J. Snape J. W. (1989). Location of a gene for frost resistance on chromosome 5A of wheat. *Euphytica* 42 41–44. 10.1270/jsbbs.63.58

[B62] Tamura K. Yamada T. (2007). A perennial ryegrass CBF gene cluster is located in a region predicted by conserved synteny between *Poaceae* species. *Theor. Appl. Genet.* 114 273–283. 10.1007/s00122-006-0430-z 17075706

[B63] Tompkins D. K. Ross J. B. Moroz D. L. (2000). Dehardening of annual bluegrass and creeping bentgrass during late winter and early spring. *Agron. J.* 92 5–9. 10.2134/agronj2000.9215

[B64] Tremblay K. Ouellet F. Fournier J. Danyluk J. Sarhan F. (2005). Molecular characterization and origin of novel bipartite cold-regulated ice recrystallization inhibition proteins from cereals. *Plant Cell Physiol.* 46 884–891. 10.1093/pcp/pci093 15792959

[B65] Trevaskis B. (2010). The central role of the VERNALIZATION 1 gene in the vernalization response of cereals. *Funct. Plant Biol.* 37 479–487. 10.1071/FP10056

[B66] Tronsmo A. M. (1985). Effects of dehardening on resistance to freezing and to infection by *Typhula ishikariensis* in Phleum pratense. *Acta Agric. Scand.* 35 113–116. 10.1080/00015128509435764

[B67] Turner A. Beales J. Faure S. Dunford R. P. Laurie D. A. (2005). The pseudoresponse regulator Ppd-H1 provides adaptation to photoperiod in barley. *Science* 310 1031–1034. 10.1126/science.1117619 16284181

[B68] Xiong Y. Fei S.-Z. (2006). Functional and phylogenetic analysis of a DREB/CBF-like gene in perennial ryegrass (*Lolium perenne* L.). *Planta* 224 878–888. 10.1007/s00425-006-0273-5 16614820

[B69] Yan L. Loukoianov A. Tranquilli G. Helguera M. Fahima T. Dubcovsky J. (2003). Positional cloning of the wheat vernalization gene VRN1. *Proc. Natl. Acad. Sci.* 100 6263–6268. 10.1073/pnas.0937399100 12730378 PMC156360

[B70] Zhang C. Fei S.-Z. Arora R. Hannapel D. J. (2010). Ice recrystallization inhibition proteins of perennial ryegrass enhance freezing tolerance. *Planta* 232 155–164. 10.1007/s00425-010-1163-4 20379831

