# Supplementary material for: Vernalization Requirement and the Chromosomal VRN1-Region can Affect Freezing Tolerance and Expression of Cold-Regulated Genes in Festuca pratensis
Source: Front Plant Sci. 2016 Feb 25;7:207. doi: 10.3389/fpls.2016.00207 (PMC4766358; doi:10.3389/fpls.2016.00207)
Supplement: Supplementary file 1 [file Table_1.DOC]

Supplementary Material

# Vernalization requirement and the chromosomal *VRN1*-region can affect freezing tolerance and expression of cold-regulated genes in *Festuca pratensis*

Åshild Ergon^*^, Tone Ingeborg Melby, Mats Höglind, Odd Arne Rognli

*** Correspondence:** Corresponding Author: ashild.ergon@nmbu.no

**Supplementary Table 1.** Gene transcripts analysed by qPCR.

| **Gene** | **Genbank acc. no. and reference** | **Primer sequences** | **Product size (bp)** |
| --- | --- | --- | --- |
| *ACTIN* | Jurczyk et al. 2012, 2013 | GTCGAGGGCAACATATGCAA  CCAGTGCTGAGCGGGAAAT | 62 |
| *VRN1* | DQ108934, Ergon et al. 2006 | CTCAAGCGGATCGAGAACA  TTCCCTTGGTGGAGAAGATG | 136 |
| *COR14B* | Rudi et al. 2011, Jurczyk et al. 2012 | AGACCCAGATCGATGGCTTCT  GCACGGCCTGGGAAGAG | 102 |
| *CR7* | Rudi et al. 2011 | CCGTTCCTTATGTGCTCCAT  CAAGAAGGCGATCACACTGA | 84 |
| *MADS3* | GU574697, Ergon et al. 2013 | GAGCAGACGAATGGAGCA  ACTGATGGTGCGGAGCAT | 237 |
| *IRI1* | Rudi et al. 2011 | TGCTTCCAACACACACACAC  CAAGAGGAACGCCAAAAAGA | 148 |
| *CBF6* | DQ996012, Rudi et al. 2011, Alm et al. 2011, Jurczyk et al. 2012 | CTTCGCAGAACGACAATTCG  GGTCCCATCCCATATCACTGA | 60 |
| *LOS2* | Rudi et al. 2011, Alm et al. 2011, Jurczyk et al. 2012 | AGATCGTAGGAGATGACCTTCTTGT  TGCAGGTCTTCTCACTGATTGC | 72 |
